# Function of KvLQT1 potassium channels in a mouse model of bleomycin-induced acute lung injury

**DOI:** 10.3389/fphys.2024.1345488

**Published:** 2024-02-20

**Authors:** Mélissa Aubin Vega, Alban Girault, Émilie Meunier, Jasmine Chebli, Anik Privé, Annette Robichaud, Damien Adam, Emmanuelle Brochiero

**Affiliations:** ^1^ Centre de recherche du Centre hospitalier de l’Université de Montréal (CRCHUM), Montréal, QC, Canada; ^2^ Département de Médecine, Université de Montréal, Montréal, QC, Canada; ^3^ Laboratoire de Physiologie Cellulaire et Moléculaire (LPCM UR UPJV 4667), Amiens, France; ^4^ SCIREQ Scientific Respiratory Equipment Inc., Montréal, QC, Canada

**Keywords:** potassium channels, acute lung injury, animal model, pulmonary inflammation, respiratory function, injury and repair, alveolar-capillary barrier

## Abstract

Acute respiratory distress syndrome (ARDS) is characterized by an exacerbated inflammatory response, severe damage to the alveolar-capillary barrier and a secondary infiltration of protein-rich fluid into the airspaces, ultimately leading to respiratory failure. Resolution of ARDS depends on the ability of the alveolar epithelium to reabsorb lung fluid through active transepithelial ion transport, to control the inflammatory response, and to restore a cohesive and functional epithelium through effective repair processes. Interestingly, several lines of evidence have demonstrated the important role of potassium (K^+^) channels in the regulation of epithelial repair processes. Furthermore, these channels have previously been shown to be involved in sodium/fluid absorption across alveolar epithelial cells, and we have recently demonstrated the contribution of KvLQT1 channels to the resolution of thiourea-induced pulmonary edema *in vivo*. The aim of our study was to investigate the role of the KCNQ1 pore-forming subunit of KvLQT1 channels in the outcome of ARDS parameters in a model of acute lung injury (ALI). We used a molecular approach with KvLQT1-KO mice challenged with bleomycin, a well-established ALI model that mimics the key features of the exudative phase of ARDS on day 7. Our data showed that KvLQT1 deletion exacerbated the negative outcome of bleomycin on lung function (resistance, elastance and compliance). An alteration in the profile of infiltrating immune cells was also observed in KvLQT1-KO mice while histological analysis showed less interstitial and/or alveolar inflammatory response induced by bleomycin in KvLQT1-KO mice. Finally, a reduced repair rate of KvLQT1-KO alveolar cells after injury was observed. This work highlights the complex contribution of KvLQT1 in the development and resolution of ARDS parameters in a model of ALI.

## 1 Introduction

Acute respiratory distress syndrome (ARDS; (ARDS Definition Task Force et al., 2012; Riviello et al., 2017; Matthay et al., 2024)) is one of the most severe forms of respiratory failure in the intensive care unit. Although the management of ARDS patients has greatly improved over the last decades, the mortality rate (over 30%–45%) remains unacceptably high and a better understanding of the physiopathology of ARDS is still needed (Bellani et al., 2016). ARDS is a complex and heterogeneous syndrome that can occur after direct (e.g., pneumonia, aspiration) or indirect (e.g., sepsis) pulmonary insult and develops in three overlapping phases described as exudative, proliferative, and fibrotic (Bos and Ware, 2022). The acute exudative phase is characterized by severe epithelial alveolar and endothelial vascular damage leading to protein-rich edema infiltration, massive neutrophil recruitment, high cytokine/chemokine levels and decreased lung compliance (Matthay et al., 2019). The emergence of collagen deposition and fibroproliferation, which compete with epithelial repair, may progress toward irreversible pulmonary fibrosis. Therefore, the resolution of the reversible exudative phase prior to the establishment of fibrosis is a key determinant of ARDS recovery and patient survival (Matthay et al., 2019). Experimental studies in animal models of acute lung injury (ALI) and clinical trials in patients with ARDS have yielded conflicting results regarding the effect of therapeutic strategies that target the inflammatory response or aimed to improve edema resolution and lung compliance (Bosma et al., 2010; Fronius, 2013; Matthay, 2014; Mokra et al., 2019; Horie et al., 2020; Silva et al., 2020). Despite extensive research, there is still no effective pharmacological treatment approved for ARDS.

The resolution of ARDS is highly dependent on the ability of the alveolar epithelium to restore its integrity and function (Berthiaume et al., 1999; Gotts and Matthay, 2014). The alveolar epithelium maintains lung homeostasis through the function of alveolar cells, which are responsible for gas exchange (by alveolar type I (ATI) cells), surfactant production (by ATII cells), restoration of epithelial integrity after injury (by ATII progenitor cells) (Barkauskas et al., 2013), and alveolar fluid control (ATI and ATII cells), which is essential to keep the alveolar spaces virtually free of fluid for efficient gas exchange. Alveolar fluid clearance (AFC) is mainly driven by an active transepithelial sodium (Na^+^) absorption through apical uptake by epithelial Na^+^ channels (ENaC) (Egli et al., 2004; Hummler et al., 1996), active Na^+^ efflux *via* the basolateral Na^+^/K^+^-ATPase pump, and subsequent potassium (K^+^) recycling by basolateral K^+^ channels (Basset et al., 1987).

K^+^ channels, of which thirty different types have been identified in the respiratory epithelium, play a critical role in lung homeostasis (Bardou et al., 2009; Girault and Brochiero, 2013). One of the main functions of K^+^ channels in epithelia is to control the membrane potential and thus to maintain an electrochemical gradient necessary for transepithelial ion and fluid transport (Markus and Qi-Xian, 2007; Bardou et al., 2009)*.* In alveolar epithelial cell cultures, K_ATP_ and KvLQT1 channels contribute to the majority of the total basolateral K^+^ currents and are involved in the control of Na^+^ absorption (by regulating the ENaC channel) and, secondarily, in fluid clearance (Leroy et al., 2004; 2006; Bardou et al., 2012). In resected human lung, Sakuma *et al.* showed that a K_ATP_ channel opener favors fluid absorption across the alveolar epithelium, by a mechanism secondarily mediated by amiloride-sensitive Na^+^ channels (Sakuma et al., 1998). Furthermore, in one of our recent studies (Aubin Vega et al., 2023), we showed that KvLQT1 knockout (KO) in mice was associated with a small but significant increase in water lung content under physiological conditions, compared to wild-type (WT) mice, while both the lung function and histological structures were generally preserved in KvLQT1-KO mice. Our data then showed that pharmacological activation of KvLQT1 in WT mice favors the resolution of thiourea-induced lung edema, suggesting a role for KvLQ1 in fluid clearance (Aubin Vega et al., 2023).

A few studies have also examined K^+^ channel function in models of ALI. Although no changes in lung structure or function were observed between WT and KO mice for TREK-1 (a mechanosensitive two-pore domain (K2P) K^+^ channel) at baseline, TREK-1-KO mice exposed to hypoxia and mechanical ventilation showed worse lung compliance, increased cellular apoptosis, but lower levels of pro-inflammatory cytokines than WT mice (Schwingshackl et al., 2014). There is further evidence that the alveolar inflammatory response *in vivo* and *in vitro* can be influenced by K^+^ channels (Schwingshackl et al., 2013a; Schwingshackl et al., 2013b; Schwingshackl et al., 2015; Schwingshackl et al., 2017; Zhou et al., 2019; Zyrianova et al., 2020; Petersen et al., 2022). For example, the role of the regulatory (β) subunit of KvLQT1 channels was investigated by Zhou *et al.* using KCNE2 knockout mice (Zhou et al., 2019), which exhibited increased levels of inflammatory mediators and altered lung function at baseline, as well as impaired response to ischemia-reperfusion injury. To our knowledge, the effect of deleting the pore-forming subunit (KCNQ1) of KvLQT1 has never been investigated in an animal model of ALI. Nevertheless, our laboratory has previously reported that the pharmacological modulation of K^+^ channels (specifically KvLQT1 and K_ATP_) affects alveolar repair processes after injury *in vitro* (Trinh et al., 2007).

Based on all this evidence, it can be postulated that K^+^ channel modulation may have an impact on the development and resolution of ARDS parameters. Therefore, the aim of our study was to evaluate the function of KvLQT1 channels on the outcome of ALI, using an animal model of severe alveolar damage. We used a molecular approach with KO mice carrying a targeted mutation in the gene encoding the pore-forming α-subunit (KCNQ1) of the KvLQT1 channel. We chose the well-characterized model of acute lung injury induced by bleomycin. Although no single animal model perfectly recapitulates all the features of ARDS in humans, the bleomycin-induced model (Izbicki et al., 2002; Matute-Bello et al., 2008) exhibits key characteristics of ARDS, according to the American Thoracic Society report (Matute-Bello et al., 2011; Kulkarni et al., 2022). Focusing on the acute exudative phase (on day 7), we compared lung function parameters, alveolar barrier function, inflammatory profile, and performed histological and immunofluorescence analyses of lung sections from WT and KO mice. Our results suggest that KvLQT1 ablation exacerbates the deleterious outcomes of bleomycin on lung function. Further analysis highlighted the complex function of KvLQT1, whose deletion is associated with changes in the profile of infiltrating immune cells and reduced interstitial and/or alveolar inflammatory response, whereas the repair capacity of KvLQT1-KO AT2 cells was slowed after injury.

## 2 Material and methods

### 2.1 Animals and ethical statements

Mice with a targeted disruption of the *kcnq1* gene (constitutive *kcnq1* ^−/−^ knock-out (KO) mice), encoding the KCNQ1 protein (pore-forming, α-subunit of the KvLQT1 K^+^ channel) were originally generated by insertion of a neomycin cassette in exon 2 by Dr. K. Pfeifer’s group (Laboratory of Mammalian Genes and Development, NICHD/National Institutes of Health, Bethesda, United States of America), as described previously (Casimiro et al., 2001). The initial breeding pairs (on a C57BL/6J background) were kindly provided by Dr. K. Pfeifer. The mouse colony was then maintained by breeding heterozygous mice (male *kcnq1*+/−x female *kcnq1*+/−) at the Centre de Recherche du Centre Hospitalier de l'Université de Montréal (CRCHUM) animal care facility and backcrossing with C57BL/6J wild-type mice (purchased from The Jackson Laboratory) every 10 generations (Aubin Vega et al., 2023). Mice were maintained in a controlled environment with *ad libitum* access to water and food (2018 Teklad global 18% protein rodent diets, Envigo, United States). Pups were ear punched at weaning for genotyping by PCR (using G-KOF (5′-CCA GGA GTG GGT GGT TCT AC - 3′), G-KONF (5′-CGC TTC CTC GTG CTT TAC G-3′) and G-KOR (5′-GCC AGC ACT AAA GAT CTT GC-3′) primers (Integrated DNA Technologies, US) amplifying 240- and 370-bp products, for WT and mutant alleles, respectively) and were recognized as WT or KO mice (Aubin Vega et al., 2023). All animal procedures were approved by the Institutional Committee for the Protection of Animals (CIPA) at the CRCHUM, in accordance with the guidelines of the Canadian Council for Animal Care (CCAC).

### 2.2 *In vivo* experimental conditions

*In vivo* experiments were performed on 6- to 10-week-old mice randomly assigned to the experimental groups described below (matched for weight and sex). WT and KO mice were compared at baseline (in control conditions, PBS) and after bleomycin-induced acute lung injury (Bleo). Bleomycin (3 U/kg, Fresenius Kabi, Canada) was administered by intranasal instillation (i.n., 50 µL) on day 0; the control group received the same volume of PBS (i.n.). This route of administration was chosen over the intratracheal technique because it is non-invasive, rapid (requiring only a few seconds of isoflurane anesthesia from which the animals regain consciousness very quickly), and spontaneous nasal aspiration allows for a more homogeneous distribution of the administered fluid throughout the lungs. The dose of 3 U/kg of bleomycin was chosen to induce severe lung injury without significant mortality. Only a negligible number of mice had to be euthanized because they had reached the endpoints and were therefore excluded from the outcome measurements. The following 4 experimental groups (genotype/i.n.) were then compared: WT/PBS, WT/Bleo, KO/PBS, and KO/Bleo, as indicated in each figure legend. On day 7, mice were anesthetized for blood gas, electrolyte and metabolite analysis, or lung function measurements, or euthanized with an overdose of pentobarbital (intraperitoneal injection, i. p.) prior to lung collection. Samples were then used for water lung content assays, histology/immunofluorescence analyses, or isolation of primary alveolar epithelial cells before primary culture and wound-healing assays (see below).

### 2.3 Measurement of blood gases, electrolytes, and metabolites with the epoc^®^ blood analysis system

On day 7, mice were first anesthetized with isoflurane (3% mixed with 21% O_2_ and medical air, to mimic environmental conditions). The abdomen was then cut open to access the inferior vena cava, and venous blood was collected using a 1 mL syringe and a 25 G heparinized needle (Fresenius Kabi). The collected blood was placed in the epoc^®^ Blood Analysis System (Siemens Healthineers Canada), which allows measurement of blood gases (pH, pCO_2_), electrolytes (HCO_3_^−^, Na^+^, K^+^, Ca^++^, Cl^−^), and metabolites (glucose, lactate, and urea). Mice were then euthanized by cervical dislocation and the lungs were snap frozen for mRNA extraction or embedded for histological analysis.

### 2.4 Measurements of lung function *in vivo*

Mechanical properties (resistance, elastance, and compliance) of the respiratory system were assessed in live WT and KO mice, challenged or not (i.n. PBS) with bleomycin (Bleo, 3 U/kg) using the flexiVent FX system (SCIREQ, Montreal, QC, Canada), as previously described (Robichaud et al., 2017; Aubin Vega et al., 2023). Briefly, mice received an administration of xylazine hydrochloride (12 mg/mL, i. p.) 5 min before being deeply anesthetized with sodium pentobarbital (70 mg/kg, i. p.). They were then tracheotomized, cannulated (18-gauge metal cannula with a typical resistance of 0.22 cmH_2_O.s/mL), and connected to a computerized small animal ventilator for mechanical ventilation. Details of flexiVent settings, data acquisition, and measurement maneuvers were as previously described in a previous study (Aubin Vega et al., 2023). Briefly, data acquisition was started after two deep lung inflations to 30 cmH_2_O, to open closed lung areas and standardize lung volumes. A total of four consecutive and different measurement perturbations were performed as one cycle, and the cycle was repeated until at least three acceptable measurements were recorded (the average of these triplicates is shown as a single dot in each of the graphs).

The mechanical properties of the respiratory system were determined by using both single (SnapShot-150) and broadband (Quick Prime-3) forced oscillation perturbations, and the overall resistance of respiratory system (*R*_*rs*_), elastance (*E*_*rs*_), and compliance (*C*_*rs*_) were obtained by fitting the classical single compartment model to the experimental signals of the single forced oscillation measurement. The constant-phase model also allowed us to determine Newtonian resistance (R_N_), tissue elastance (*H*), and tissue damping (*G*) parameters from the broadband forced oscillation signals (Quick Prime-3).

Pulmonary compliance was characterized using pressure-volume (PV) curves constructed by inflating the lungs with pressure increments from PEEP to 30 cmH_2_O and then deflating in a similar manner. The deflation limbs were analyzed as previously described (Robichaud et al., 2021) and used to determine the quasi-static compliance (*C*_*st*_).

### 2.5 Pulmonary edema index

After euthanasia on day 7, the vena cava was cut; the lungs were removed and weighed directly (wet weight) (Aubin Vega et al., 2019; Aubin Vega et al., 2021; Aubin Vega et al., 2023). The lungs were then heated at 95°C for 24 hours to measure the dry weight. The water content of the lungs, as an index of pulmonary edema, was then calculated using the following formula (Kabir et al., 2002):

$$WLC\;\left(mg/g\right)=\left(wet\;weight\;–\;dry\;weight\right)\;/\;mice\;weight$$

### 2.6 Evans blue extravasation assay

The Evans Blue extravasation assay, a commonly used technique to evaluate pulmonary permeability after endothelial damage, was performed on mice (WT and KO) under control conditions (WT/PBS and KO/PBS) or 7 days after bleomycin challenge (Bleo, 3 U/kg). Briefly, a solution of Evans Blue (50 mg/kg, Sigma) was injected into the tail vein (intravenous injection, i. v.), and after 3 hours of blood circulation, the mice were euthanized; the lungs were perfused with PBS-EDTA (5 mM, 1 mL) *via* the pulmonary artery and then washed with subsequent baths of PBS, as previously described (Aubin Vega et al., 2023). Lungs were minced with scissors and incubated with formamide (Sigma) at 37°C for 18 h. The resulting homogenate was centrifuged and the luminescence of the supernatant was measured at 620 and 740 nm to determine the concentration of Evans Blue according to a standard curve. The results were then corrected for heme pigment using the following formula:

$$E620\;\left(EBD\;corrected\right)=E620\;–\;\left(1.426\;x\;E740+0.030\right)$$

### 2.7 Analysis of broncho-alveolar lavage (BAL) fluid

In another set of experiments, BALs were obtained after mouse euthanasia on day 7 post-bleomycin challenge by instillation of saline (1 mL) through a catheter. Three replicate BALs (from the same mouse) were pooled on ice prior to centrifugation (700 g, 4°C, 8 min). Supernatants were aliquoted and stored at −80°C until used to determine protein concentration by the Bradford method (Bio-Rad Life Science, Mississauga, ON, Canada).

Cell pellets were resuspended in PBS (400 μL) for total immune cell count quantification in a hemocytometer. Cell suspensions were then diluted to a density of 80,000 cells (in 200 μL PBS/slide) before cytocentrifugation (750 rpm, 5 min, Thermo Scientific Cytospin 4 Cytocentrifuge, Block Scientific, NY, United States) onto glass slides and staining with Hema 3™ Stat Pack (Fisher Healthcare, United States). The immune differential cell count was then determined and reported as the percentage of neutrophils, macrophages and lymphocytes among a total of 400 leukocytes/slide counted.

### 2.8 RNA extraction and real-time quantitative PCR

After harvesting, the lungs were immediately snap frozen in 2-methylbutane on dry ice and stored at −80°C until needed. One of the lungs was crushed in liquid nitrogen and the total RNA was extracted using RNeasy mini kits (QIAGEN, Toronto, Canada). RNA concentration and purity were assessed using a NanoDrop™ One spectrophotometer (Thermo Fisher Scientific, Madison, United States). Samples without OD_260/280_ ≥ 1.8, OD_260/230_ ≥ 1.8, were disqualified. RNA integrity was also verified by migrating the samples onto an agarose gel (1%) containing SYBR Safe DNA Gel Stain (Invitrogen, Carlsbad, Canada) and visualized with the Typhoon Gel Imager (Typhoon TRIO variable mode imager, General Electric Healthcare). RNA extracts were then treated with DNase (DNA-*free*™ DNA Removal Kit, Invitrogen) before reverse transcription of RNA (1 µg) to cDNA using the iScript Reverse Transcription SuperMix Kit (Bio-Rad, Hercules, CA, United States) and the DNA Thermal Cycler (Perkin Elmer Cetus, Woodbridge, Canada).

For PCR amplification and quantitative analysis of TNF-α, IL-1β, IL-6, KC (the murine equivalent of human IL-8), and MCP-1 gene expression levels, 1.25–50 ng of cDNA, depending on the dilution factor specific to each gene of interest, was amplified using the SsoAdvanced universal SYBR Green Supermix Kit (Bio-Rad) with the QuantStudio™ 5 System (Thermo Fisher Scientific) in the presence of 337.5 nM of forward and reverse primers designed on the National Center for Biotechnology Information Primer Blast website and synthesized by Integrated DNA Technologies Canada based (see sequences in Table 1). All kits were used according to the manufacturer’s instructions. The delta-delta Ct method (2^−ΔΔCT^ method) was used to calculate the relative expressions compared to the control groups, and the expression level of the housekeeping gene β-actin was used for normalization. Data are expressed as log_2_ (∆∆Ct).

**TABLE 1:** Primer sequences and RT-qPCR conditions.

Gene	Forward primer	Reverse primer	Cycling conditions
TNF-α	5′-CCC CAA AGG GAT GAG AAG TT-3′	5′-CAC TTG GTG GTT TGC TAC GA-3′	95°C–5min 40x (95°C - 15s +60°C - 30 s)
IL-1β	5′-GTT GAC GGA CCC CAA AAG-3′	5′-GTG CTG CTG CGA GAT TTG-3′	
IL-6	5′-TCT CTG GGA AAT CGT GGA A-3′	5′-TCT GCA AGT GCA TCA TCG T-3′	
KC	5′-TGA AGC TCC CTT GGT TCA G-3′	5′-GGT GCC ATC AGA GCA GTC T-3′	
MCP-1	5′-AGC TGT AGT TTT TGT CAC CAA GC-3′	5′-GAC CTT AGG GCA GAT GCA GT -3′	
β-actin	5′-TTG CTG ACA GGA TGC AGA AG-3′	5′-ACA TCT GCT GGA AGG TGG AC-3′	

### 2.9 Histological analysis

To prevent alveolar collapse, a fixative solution of 4% paraformaldehyde (500 μL, Electron microscopy sciences, United States) was carefully administered by intratracheal instillation (i.t.) prior to lung harvesting. Tissue samples were incubated in a sucrose gradient (24 h for each gradient of 5%, 10%, and 20%) in a solution of paraformaldehyde (0.4%) and embedded in resin (Shandon Cryomatrix, Thermo Fisher Scientific). After cryosectioning (5 μm) with a cryostat device (Leica Microsystems, model CM1950110111), slides were stained with hematoxylin and eosin (Rapid-Chrome Frozen Sections Staining Kit, ThermoScientific, United States) and scanned with a Versa stand at ×20 objective on a Leica^®^ light microscope, prior to histological analysis or frozen until immunostaining.

We first established a lung injury score by computational analysis using Visiomorph™ software (Visiopharm, Hoersholm, Denmark), based on septal thickening, the presence of fibrotic foci, and immune cell infiltration throughout the lung section to account for the heterogeneous nature of inflammatory damage with both intact and injured/inflamed areas. The closer the injury score is to 1, the more damaged and congested the lungs are, altering gas exchange capacity. A team of pathologists (Drs. Feryel Azzi and Guillaume St-Jean from the Molecular Pathology Platform at CRCHUM) also performed a blinded histological analysis to quantify the percentage of total area with diffuse alveolar damage and immune cell (polynuclear neutrophil) infiltration (expressed as % of injured/inflamed area out of the total area of the lung section). This lung injury score was adapted from the official American Thoracic Society workshop report (Matute-Bello et al., 2011; Kulkarni et al., 2022), a well-established scoring system for experimental models of ALI, and takes into account of overall (interstitial and alveolar) inflammation, alveolar wall thickening (mainly due to immune cell infiltration), and the presence of debris. The severity of inflammation in the damaged/inflamed areas (on a score of 0–3) was also defined by the team of pathologists.

### 2.10 Pro-SPC and AQP5 immunostainings of cytomatrix-embedded frozen lung sections

Lung sections (5 μm) were fixed in 0.4% paraformaldehyde before membrane permeabilization with 0.1% Triton X-100 and blocked for 1 h with a solution of PBS +10% FBS (Saradigm, United States) + 10% BSA (Sigma-Aldrich). Slides were then incubated overnight at 4°C with rabbit polyclonal anti-pro-SPC (1:100, #AB3786 Millipore, United States) or anti-AQP5 (1:100, #AQP005, Alomone Labs, Israel) antibodies. The next day, lung tissues were again blocked before incubation with an Alexa Fluor™ 568-conjugated donkey anti-rabbit secondary antibody (1:200, Life Technologies, United States) for 1 h, followed by a DAPI nuclear staining (1:1,000, Sigma) before mounting with Prolong^®^ Gold (Invitrogen, Thermo Fisher Scientific). Images were captured using an Exiqua camera (QImaging, Canada) under an inverted fluorescence microscope (Olympus, Canada) at 200x (NA = 0.75) and analyzed using ICY software (version 2.4.2.0, license GPLv3, developed by Institut Pasteur and France-BioImaging (de Chaumont et al., 2012)). Our analysis protocol allows a quantitative measurement of the intensity (in pixels) of the specific signal of each region of interest (ROI) (stained with each specific primary antibody), normalized to the total number of DAPI-positive cells (more than 24,000 cells were analyzed for each staining). This ICY protocol also allows for adjustment of the ROI detection parameters to eliminate any potential background signal. Our controls showed no background, confirming the specificity of all primary and secondary antibodies used in our assays.

### 2.11 Isolation and primary culture of mice alveolar type II (ATII) epithelial cells

ATII cells were isolated from the lungs of WT and KO mice under control conditions. Lungs from approximately 1–3 WT or KO mice were pooled to increase cell yield. After harvest, the lungs were rinced with physiological saline solution to remove excess blood cells and most of alveolar macrophages. The lungs were digested with elastase (30 U/mouse, Worthington Biochemical, Lakewood, N.J. United States, 30–45 min), minced, and then the resulting cell suspension was filtered. Alveolar epithelial cells were then purified using a differential adherence technique (Dobbs, Gonzalez, and Williams, 1986), which allows the discarding of remaining macrophages and fibroblasts attached to Petri dishes coated with IgG and then the collection of an epithelial cell suspension, enriched for up to 86% of AT2 (Brochiero et al., 2004; Leroy et al., 2004; Tan et al., 2020). Trypan blue staining of the post-IgG cell suspension confirmed >90% cell viability. After cell counting, the cell pellet was resuspended in DMEM (Gibco, United States) supplemented with 2 mM L-glutamine (Invitrogen, Ontario, Canada) and 50 U/mL penicillin-streptomycin (ThermoScientific) + 10% FBS (Saradigm, US) and seeded at a density of ∼2M cells/cm^2^ density on cell culture plates (48-well Costar, Corning Incorporated, United States) coated with laminin (Millipore) for primary culture (with medium replacement on day 2), until adequate confluence for wound-healing assays on day 4 (see below). Using this protocol, we previously showed that by day 3–4 of primary 2D culture, ∼75–85% of the cells exhibited an AT2 phenotype, while 20%–25% transdifferentiated from AT2 to AT1-like cells (Tan et al., 2020).

### 2.12 Wound-healing assays

Wound-healing assays were performed on primary cultured, confluent mouse ATII cells. Briefly, alveolar cell monolayers were mechanically injured (as per two wounds per well, two wells per conditions) with a 10 μL pipette tip according to a highly reproducible technique (Trinh et al., 2007; 2012; Girault et al., 2015; Ruffin et al., 2016; Adam et al., 2018; Aubin Vega et al., 2019). After injury, the monolayers were washed with DMEM-FBS to remove detached, injured cells. A mark under the wells allowed us to photograph with a Nikon camera under light microscopy the wounds at the same location (at time 0 and after 6 h of repair). The rate of wound closure, expressed in μm^2^/h, was calculated using ImageJ software (National Institutes of Health, Bethesda, MD, United States) from the wound area measured after repair (t = 6 h) compared to the initial wound area (t = 0 h) for each wound (as shown in Supplementary Figure S1). This time point correspond to approximately 50% of repair, which allow to observe either stimulatory or inhibitory effects on the repair capacity as a function of the experimental conditions, while wounds are generally fully repaired at 24 h (Supplementary Figure S1).

This wound healing assay provides insight into the initial repair processes engaged after injury (mainly cell migration and proliferation).

### 2.13 Statistical analyses

Data are presented as dot plots with mean ± standard error of the mean (SEM). Graphs and statistical analyses were performed using GraphPad Prism version 8 for Windows (GraphPad Software, San Diego, CA, United States). Normality tests (Agostino/Pearson) were performed first, followed by appropriate statistical tests, as described for each figure legend. Differences were considered significant when *p <* 0.05.

## 3 Results

### 3.1 Evaluation of blood parameters in mice after acute lung injury induced by bleomycin

**FIGURE 1:**
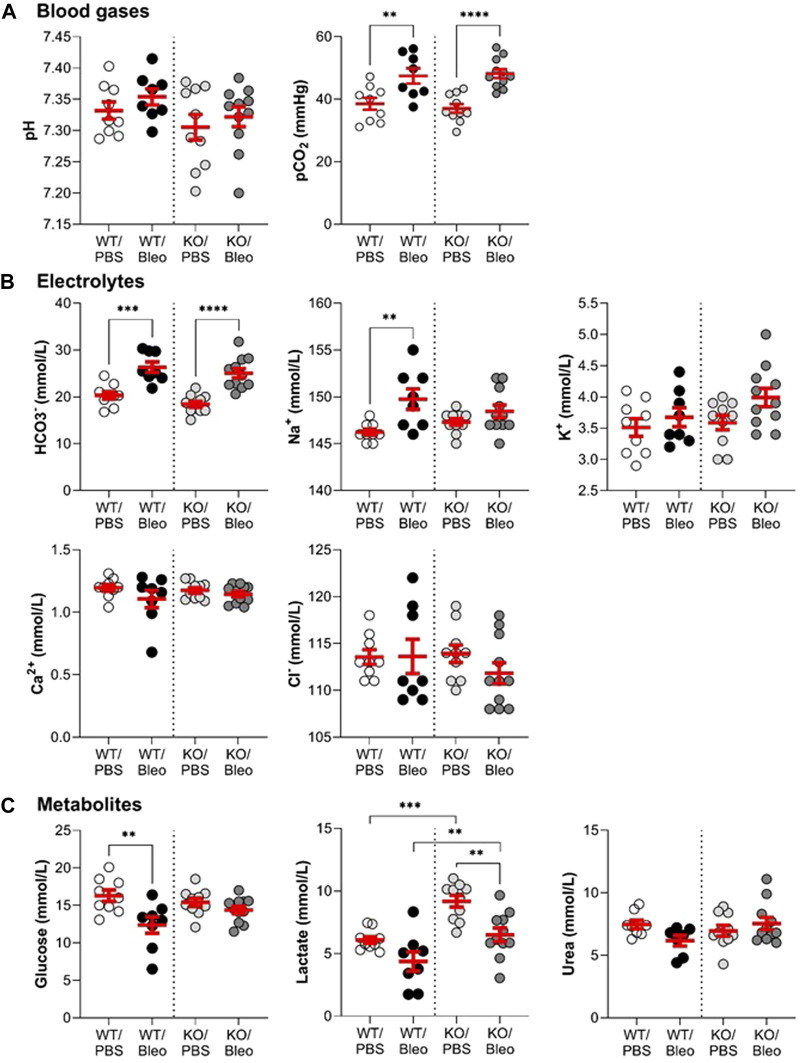
Assessment of the pulmonary and systemic dysfunction after the bleomycin challenge. Wild-type (WT, n = 8-9, shown on the left side of vertical dotted line) and KvLQT1-KO (KO, n = 10–11, right side) mice were challenged or not (PBS) with bleomycin (3 U/kg, 50 μL, **(I)** n. on day 0). On day 7, blood gas **(A)**, electrolyte **(B)**, and metabolite **(C)** levels were measured using the epoc^®^ system. Each dot represents 1 mouse and values are mean ± SEM. One-way ANOVA test (Agostino/Pearson normality test: positive) was performed for all parameters. **p < 0.01, ***p < 0.005, ****p < 0.001.

Blood analysis (Figure 1) using the epoc^®^ system was first performed to evaluate blood gases (A), electrolytes (B), and metabolites (C) in WT and KvLQT1-KO mice. Under physiological conditions, KvLQT1 deletion (KO/PBS group) did not alter blood parameters, except for an increase in lactate levels, compared to the WT/PBS group. On day 7 of the exudative phase of bleomycin-induced ALI, we observed an increase in pCO_2_ levels, which were similar in WT/Bleo and KO/Bleo mice. In both groups, we also noticed an increase in bicarbonate (HCO_3_^−^), which probably acted as a buffering system, as indicated by the stable pH values, despite the increased pCO_2_. Sodium (Na^+^) was the only electrolyte whose showed an increase after bleomycin exposure (in the WT mice only). Among the metabolites, glucose concentration was decreased in WT mice exposed to bleomycin, whereas no change was observed in KO/Bleo mice compared to KO/PBS. Bleomycin also induced a small decrease in lactate, although levels remained significantly higher in the KO/Bleo group than in the WT/Bleo group.

### 3.2 *In vivo* measurement of lung function in WT and KvLQT1-KO mice exposed to bleomycin

**FIGURE 2:**
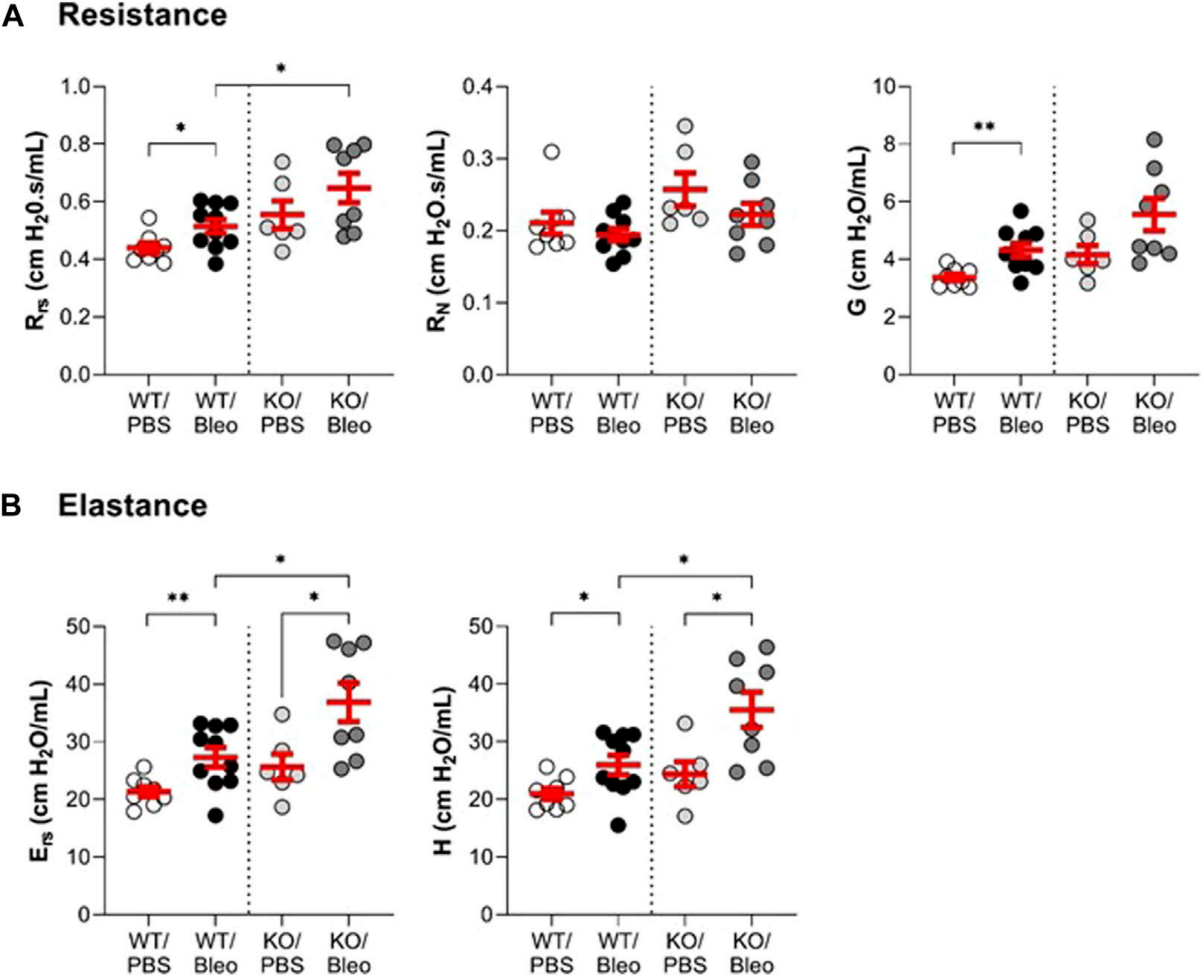
Evaluation of the lung function *in vivo* (resistance and elastance). Wild-type (WT, n = 8–10, left side of vertical dotted line) and KvLQT1-KO (KO, n = 6-8, right side) mice were challenged or not (PBS) with bleomycin (3 U/kg, 50 μL, i. n. on day 0). On day 7, several respiratory mechanics parameters reflecting lung resistance (R_rs_, R_N_ and G, panel **(A)** and elastance E_rs_ and H, panel **(B)**) were measured using the flexiVent system (SCIREQ Inc., Montreal, QC, Canada). Each dot represents 1 mouse and values are mean ± SEM. One-way ANOVA test (Agostino/Pearson normality test: positive) was performed for all parameters. *p < 0.05, **p < 0.01.

**FIGURE 3:**
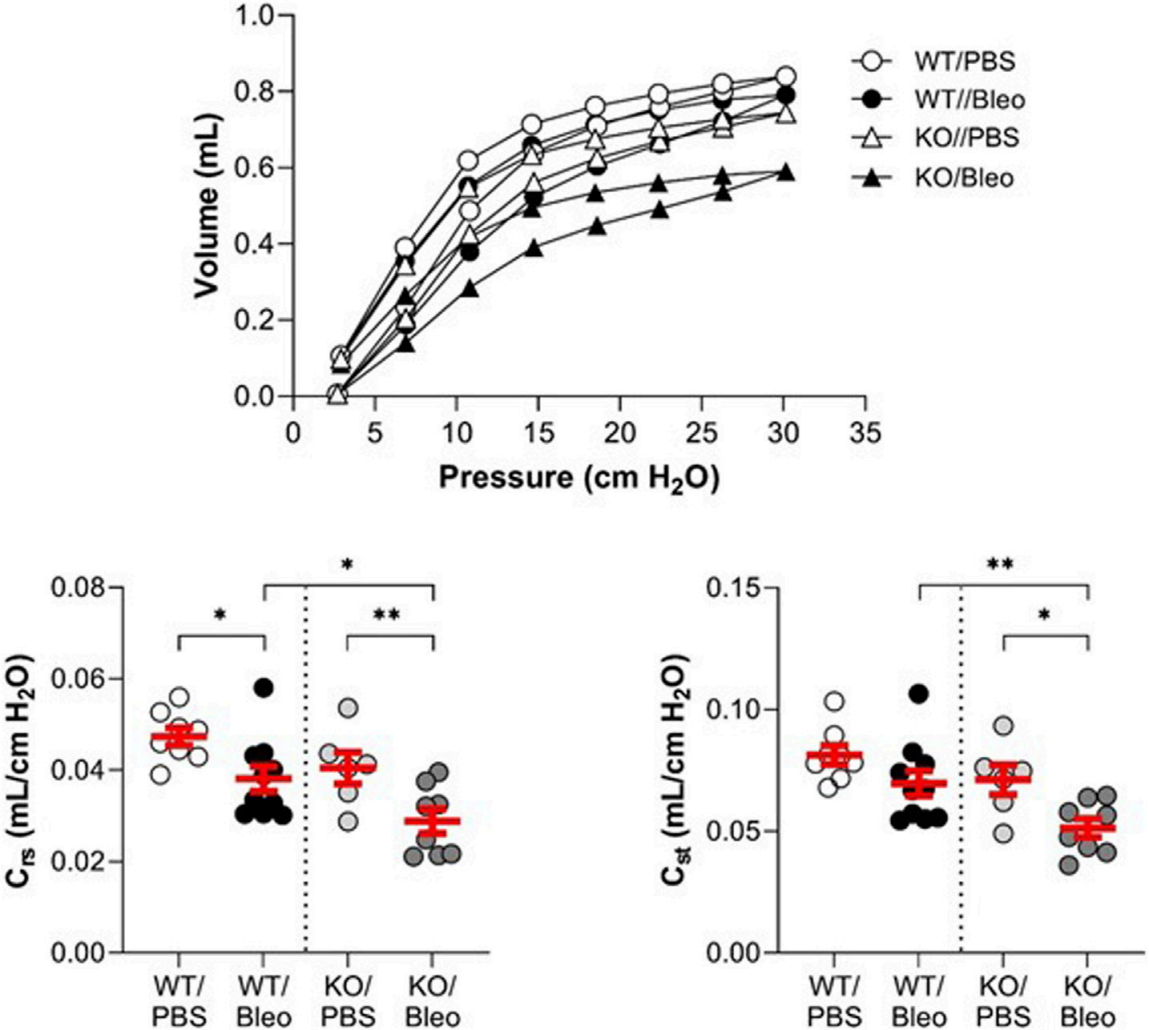
Evaluation of the lung function *in vivo* (compliance). Wild-type (WT, n = 8–10, left side of vertical dotted line) and KvLQT1-KO (KO, n = 6-8, right side) mice were challenged or not (PBS) with bleomycin (3 U/kg, 50 μL, i. n. on day 0). On day 7, several respiratory mechanic parameters reflecting lung compliance (PV loop, C_rs_ and C_st_) were measured using the flexiVent system. Each dot represents 1 mouse and values are mean ± SEM. One-way ANOVA test (Agostino/Pearson normality test: positive) was performed for all parameters. *p < 0.05, **p < 0.01.

Pulmonary function (Figure 2; Figure 3) was assessed using various measurement manoeuvers designed to characterize the biophysical properties of the lung (resistance: R_rs_, R_N_, G, elastance: E_rs_, H, or compliance: pressure-volume curve, C_rs_, C_st_) and provide information on the contribution of the whole respiratory system (R_rs_, E_rs_, C_rs_), the peripheral tissues (G, H) or the large conducting airways (R_N_).

Under physiological conditions, a non-statistically significant trend toward higher resistance and elastance and lower compliance was measured in KO/PBS mice compared to WT/PBS (Figure 2; Figure 3).

In WT mice exposed to bleomycin, the results showed a significant rise in the overall resistance of the respiratory system (R_rs_) and parameter G (which reflects energy loss during the respiratory manoeuver) compared to the WT/PBS group (Figure 2A). Newtonian resistance (R_N_) was unaffected, suggesting that the increase in R_rs_ was due to changes at the level of peripheral tissues and small airways. A similar pattern was observed in KO mice, but in this group the differences in R_rs_ and G did not reach the level of statistical significance. Nevertheless, the KO/Bleo mice had a higher respiratory resistance (R_rs_) than the WT/Bleo mice (Figure 2A, left panel).

Compared to PBS treatment, the overall elastance of the respiratory system (E_rs_) and the tissue stiffness parameter (H) were also significantly increased after bleomycin administration in both the WT and KO mice (Figure 2B). However, the deleterious effect of bleomycin was more pronounced in the KO mice than in their WT littermates.

Under physiological conditions, the pressure-volume curves showed a slight downward shift in the KO/PBS group compared to the WT/PBS group (Figure 3). This observation was also reflected by a slight decrease in the compliance parameters (C_rs_, C_st_), but without a statistically significant difference between the two groups.

In WT mice, bleomycin treatment was associated with a slight shift in the PV loop, a statistically significant decrease in dynamic compliance (C_rs_) and a non-significant trend toward a decrease in static compliance (C_st_). As also shown in Figure 3, bleomycin had a more pronounced effect in the KO mice. Indeed, the PV loop was markedly shifted downward in KO/Bleo mice with significantly lower C_rs_ and C_st_ values in KO/Bleo compared to PBS treatment (KO/PBS) as well as to the WT/Bleo group (Figure 3).

### 3.3 Bleomycin-induced alteration of the alveolar-capillary barrier

**FIGURE 4:**
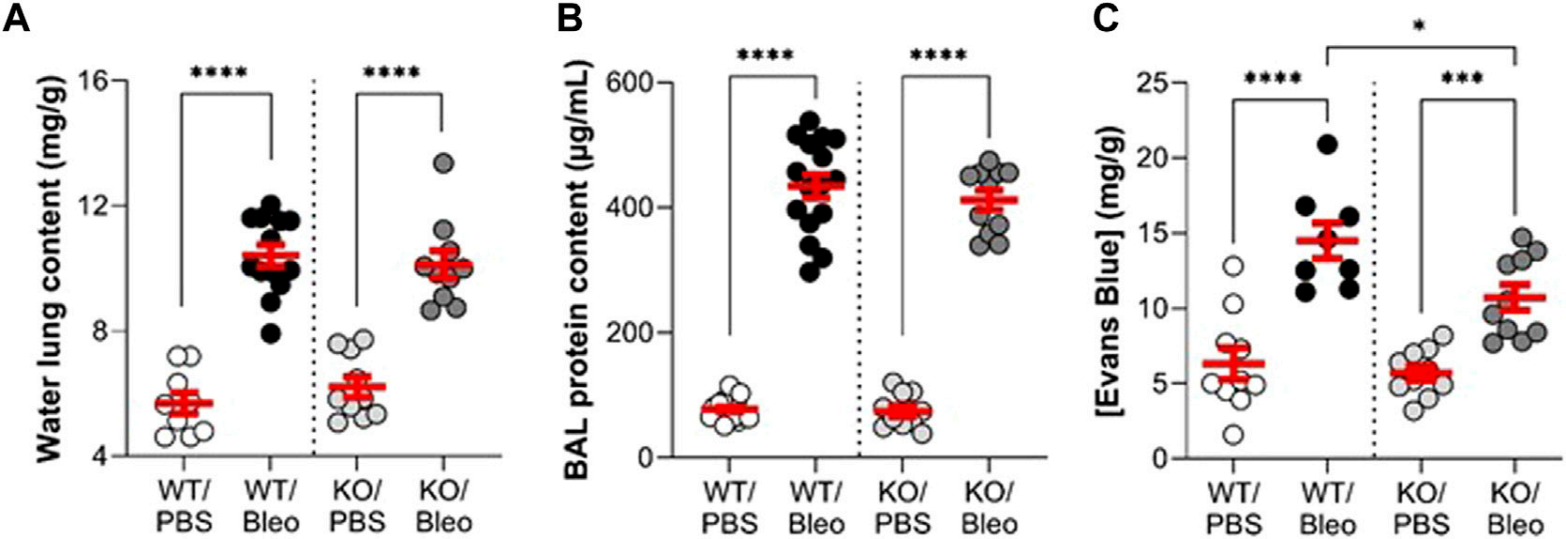
Alteration of the alveolar-capillary barrier by bleomycin in WT and KvLQT1-KO mice. Wild-type (WT, left side of vertical dotted line) and KvLQT1-KO (KO, right side) mice were challenged or not (PBS) with bleomycin (3 U/kg, 50 μL, i. n. on day 0). On day 7, water content of the lungs **(A)**, (n = 9–13), protein content in broncho-alveolar lavages (BALs) **(B)**, (n = 10–20) and Evans Blue concentration **(C)**, (n = 8–10) were measured. Each dot represents 1 mouse and values are mean ± SEM. One-way ANOVA test (Agostino/Pearson normality test: positive) for all parameters was performed for **(A–C)**. *p < 0.05, ***p < 0.005, ****p < 0.0001.

To evaluate alveolar-capillary barrier function (Figure 4), we measured water lung content (as an edema index), protein concentration in broncho-alveolar lavage (BAL), and Evans Blue extravasation in the lung compartment (interpreted as an index of endothelial permeability).

Under basal conditions, WT and KO mice showed no difference in these three parameters. Bleomycin exposure induced a twofold increase in water lung content (Figure 4A), as well as a high rise in protein concentration in BAL (Figure 4B), indicating flooding of protein-rich edema, similar in the WT and KO mice. The concentration of Evans Blue, measured after the extravasation of the dye from the blood into the lung compartment of WT/Bleo and KO/Bleo mice (Figure 4C), indicated an alteration in endothelial integrity after bleomycin, although the levels were slightly less compromised in KO than in WT mice.

### 3.4 Modulation of bleomycin-induced inflammatory response by KvLQT1 extinction

**FIGURE 5:**
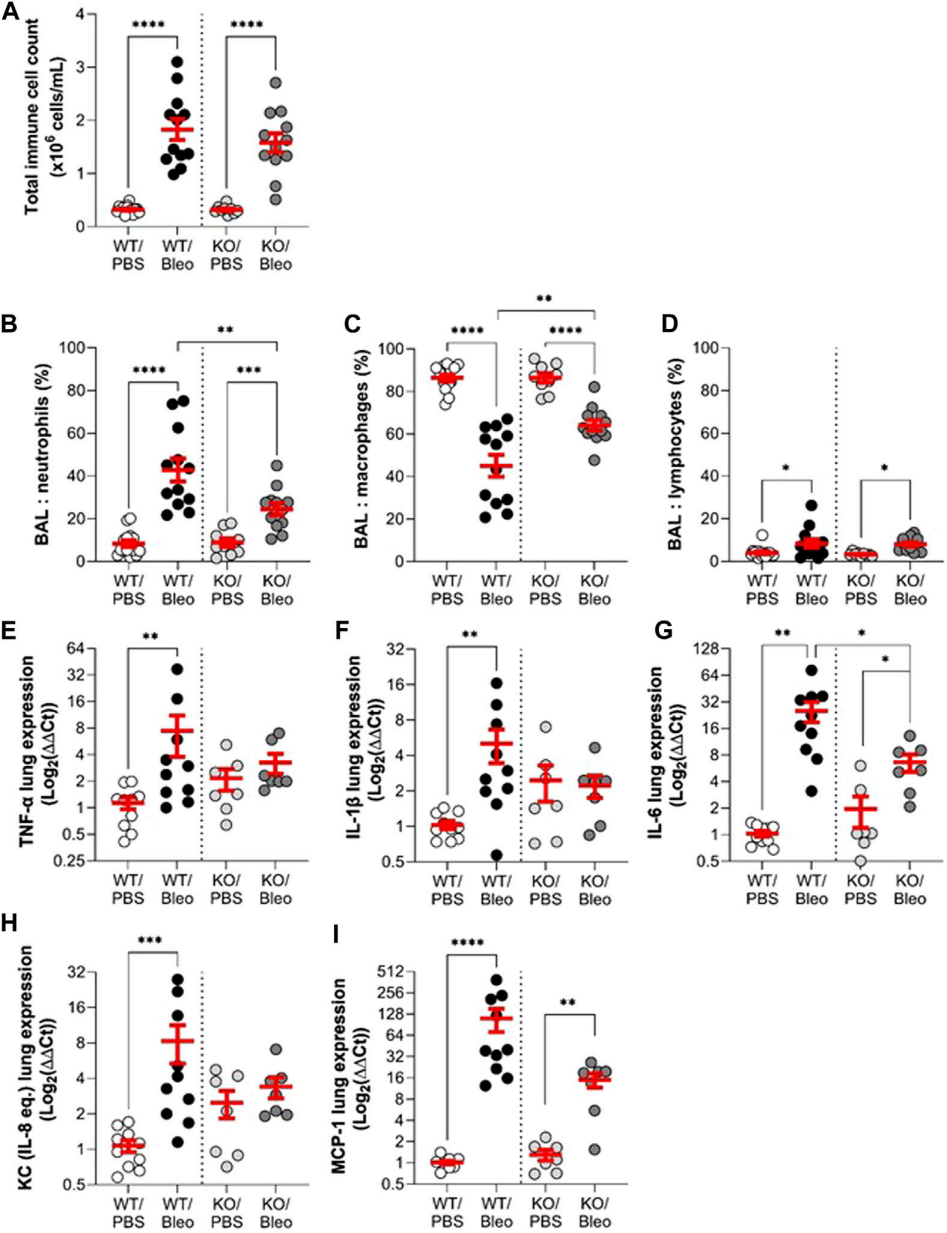
Effect of KvLQT1 modulation on bleomycin-induced inflammatory response. Wild-type (WT, left side of vertical dotted line) and KvLQT1-KO (KO, right side) mice were challenged or not (PBS) with bleomycin (3 U/kg, 50 μL, i. n. on day 0). On day 7, BAL (n = 9–14) was collected and total immune cell count was performed **(A)**. The cell pellet was then cytocentrifuged and stained with hematoxylin-eosin to obtain differential cell counts (in %) of neutrophils **(B)**, macrophages **(C)** and lymphocytes **(D)**. Pro-inflammatory cytokine **(E)**: TNF-α, **(F)** IL-1β, **and (G)** IL-6 and chemokine **(H)**: KC and **(I)** MCP-1 mRNA fold change expression (normalized to β-actin housekeeping gene) was detected from lung tissue of WT (n = 10) and KO (n = 7) mice exposed or not to bleomycin on day 7 and presented as log_2_ (∆∆Ct). Each dot represents 1 mouse and values are mean ± SEM. One-way ANOVA test (Agostino/Pearson normality test: positive) was performed for **(A–C and F)**. Kruskal–Wallis nonparametric test (Agostino/Pearson normality test: negative) was performed for **(D,E,G and H)**. *p < 0.05, **p < 0.01, ***p < 0.001, ****p < 0.0001.

The inflammatory response, another important criterion in ARDS/ALI, was first evaluated by assessing the immune cell composition in BAL (Figures 5A–D) and then the gene expression levels of pro-inflammatory cytokines/chemokines in whole lung tissue (Figures 5E–I) collected from WT and KO mice under physiological conditions as well as after bleomycin challenge (day 7).

The results of the BAL analyses showed that the immune cell composition (total cell count and percentage of neutrophils, macrophages and lymphocytes) was the same in the KO/PBS and WT/PBS controls. Under bleomycin conditions, WT and KO mice showed a similar significant increase in the total number of immune cells compared to their respective controls (PBS). However, the immune cell profile in response to bleomycin differed between WT/Bleo and KO/Bleo mice. Although bleomycin exposure caused an increase in neutrophils in both WT/Bleo and KO/Bleo mice (and a concomitant decrease in macrophage levels), a neutrophil/macrophage ratio of 43/45 was calculated in the BAL of WT/Bleo vs. 25/64 in KO/Bleo. The increase in lymphocytes was similar in WT/Bleo and KO/Bleo mice (Figures 5B–D).

We then measured the expression levels in WT and KO lungs of a panel of cytokines and chemokines, based on the inflammatory profile found in two different cohorts of ARDS patients (Meduri et al., 1995a; Goodman et al., 1996). Under basal conditions, we observed a trend for KO mice to have higher levels of most of the pro-inflammatory markers tested (except MCP-1) than WT mice, although there was no statistical difference. An increase in each of the pro-inflammatory cytokines/chemokines (TNF-α, IL-1β, IL-6, KC, and MCP-1) was measured on day 7 after bleomycin exposure in WT mice (WT/Bleo). In KO mice, however, the inflammatory response to bleomycin was impaired, with only IL-6 and MCP-1 being significantly upregulated, while TNF-α, IL-1β, and KC remained at a similar expression levels as in the control group (KO/PBS) (Figures 5E–I).

### 3.5 Evidence of bleomycin-induced acute lung injury and inflammation in histological sections

**FIGURE 6:**
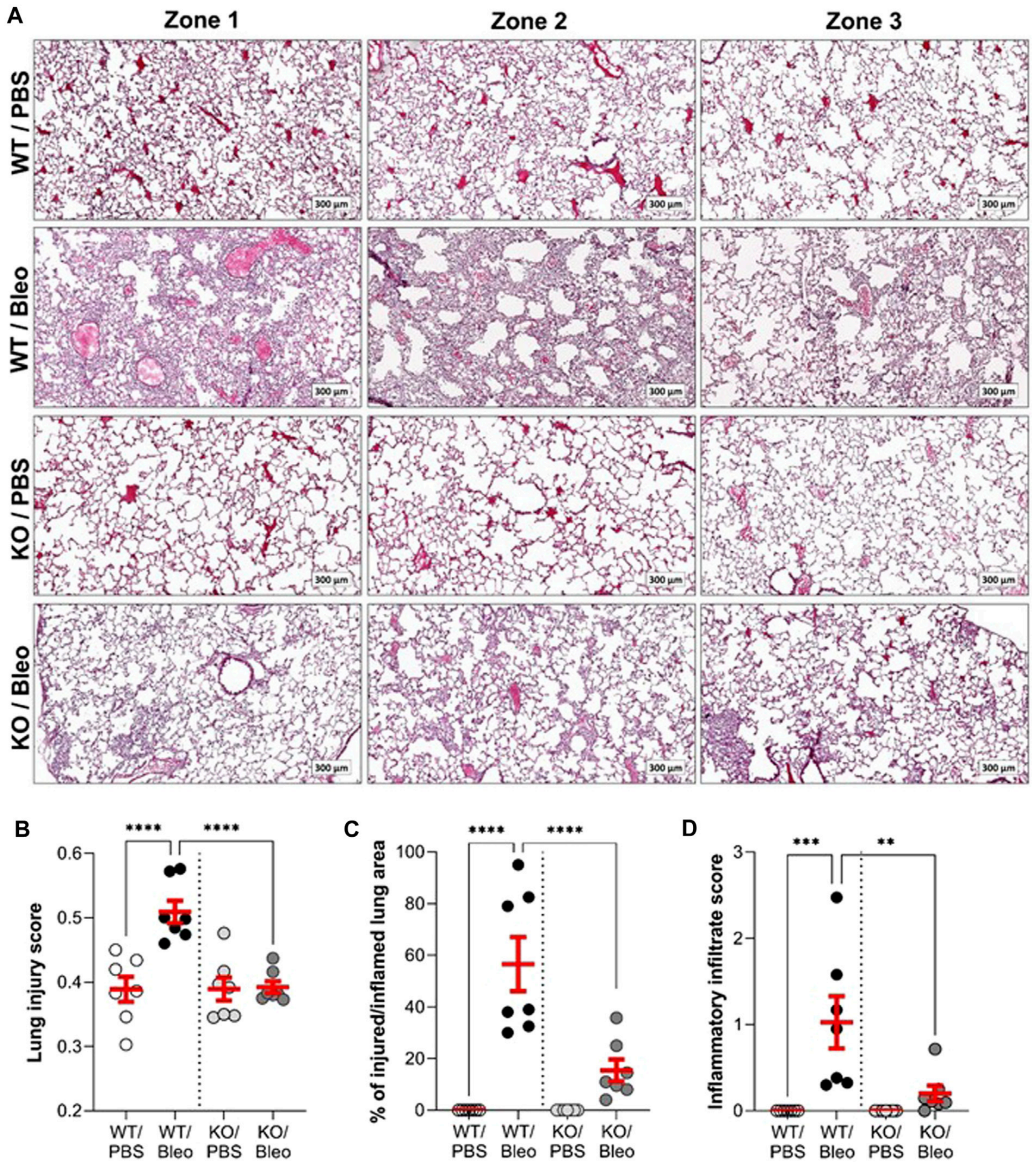
Histological evidence of bleomycin-induced inflammatory tissue damage. On day 7, fixed lung tissues from wild-type (WT, n = 7) and KvLQT1-KO (KO, n = 7) mice challenged or not (PBS) with bleomycin (3 U/kg, 50 μL, i. n. on day 0) were collected for further hematoxylin-eosin staining. Three representative zones (scale: 300 µm) from each experimental group are shown in **(A)**. A lung injury score representing the proportion of septa responsible for gas exchange **(B)** was defined using the Visiomorph^®^ software. The percentage (%) of injured/inflamed lung area **(C)** with inflammatory damage was also evaluated on the whole histological sections. The score of inflammatory infiltration intensity was also defined by pathologists from the CRCHUM molecular platform **(D)**. Each dot represents 1 mouse and values are mean ± SEM. One-way ANOVA test (Agostino/Pearson normality test: positive) was performed for **(B,C, and D)** **p < 0.01, ***p < 0.001, ****p < 0.0001.

Observations of histological lung sections (Figure 6A) showed that the WT and KO mice studied had a healthy alveolar structure under physiological conditions, as observed in our previous study (Aubin Vega et al., 2023). Intranasal administration of bleomycin induced severe changes, particularly inflammation, in the lungs of WT mice. Images of representative zones are presented (Figure 6A), showing dense congestion and cellular infiltration in zone 1, and significant septal thickening with alveolar structural changes in zone 2. The zone 3 image well illustrates the heterogeneous nature of lung damage within the same animal, with areas of extensive lung damage/inflammation juxtaposed with healthy alveolar zones. Compared to WT/Bleo, the alveolar lung architecture in KO/Bleo mice was less affected, as shown in representative images (Figure 6A) and by injury scoring (Figure 6B), based on the assessment of diffuse alveolar damage, specially the presence of an inflammatory infiltrate in the alveoli and interstitium (with septal wall thickening), as well as cellular debris, throughout the lung section (which included both intact and injured/inflamed areas). Consistent with the representative images in Figure 6A, lung injury scores (Figure 6B), percentage of injured/inflamed lung area (Figure 6C), and inflammatory infiltrate scores (Figure 6D), as defined by the pathologists, were significantly lower in KO/Bleo mice than in WT/Bleo mice.

### 3.6 Evaluation of the epithelial integrity with ATII (pro-SPC) and ATI (AQP5) cell markers

**FIGURE 7:**
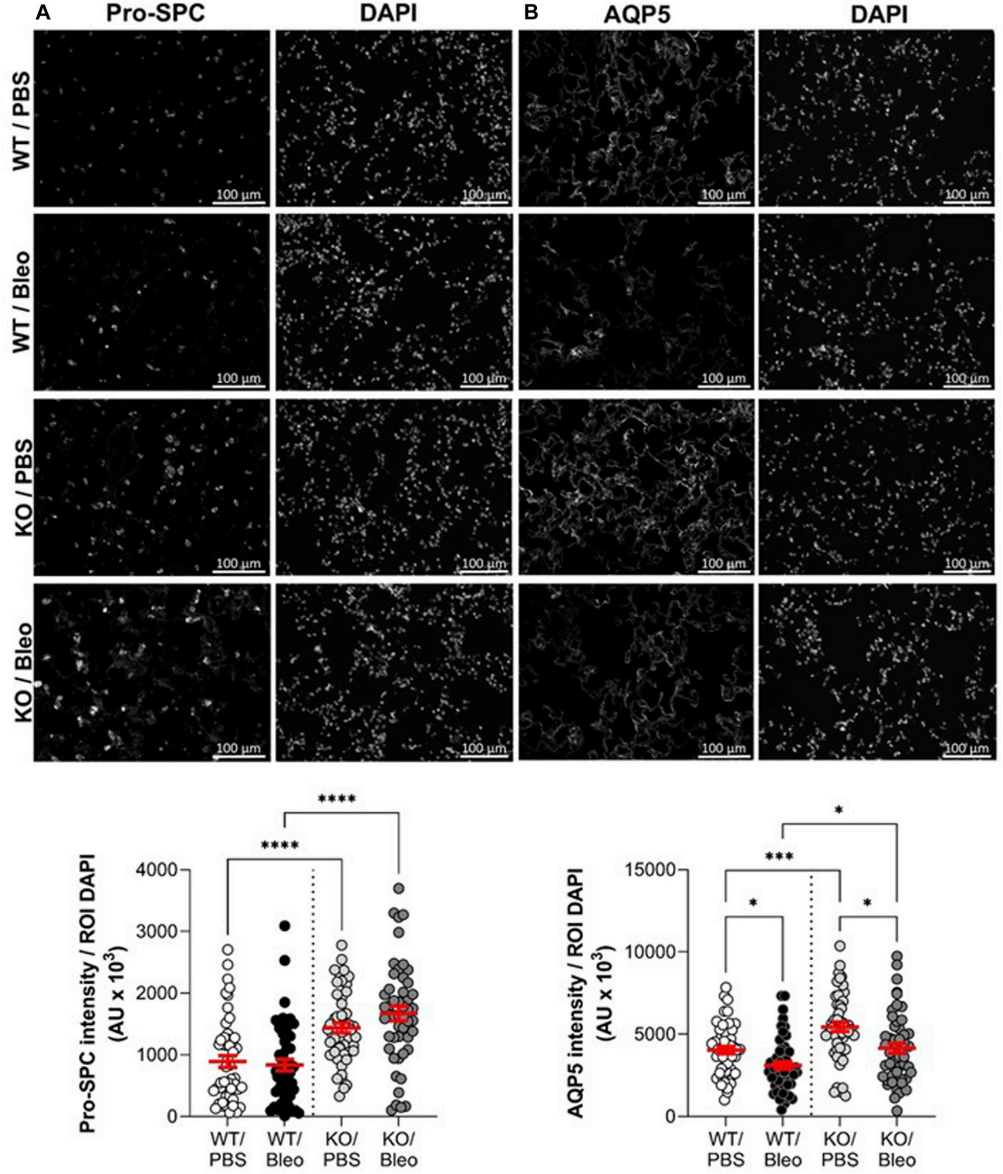
Assessment of alveolar epithelial markers after bleomycin challenge. Representative immunofluorescence images of lung sections (scale: 100 µm) from WT and KvLQT1-KO mice challenged or not (PBS) with bleomycin (3 U/kg, 50 μL, **(I)** n. on day 0). On day 7, lung tissues were fixed and immunostained for pro-SPC **(A)**, n = 50 fields, ATII marker and AQP5 **(B)**, n = 50 fields, ATI marker. Cell nuclei were stained with DAPI. Quantification of marker intensity was performed using a protocol exploited by ICY software. Values are mean ± SEM. One-way ANOVA test (Agostino/Pearson normality test: positive) was performed for **(A,B)** *p < 0.05, **p < 0.01, ***p < 0.001, ****p < 0.0001.

Because the injury score is mainly based on apparent (interstitial and alveolar) inflammation as well as alveolar wall thickening due to immune cell infiltration within the interstitium and does not specifically capture the damage to the alveolar epithelium, we next performed complementary immunostaining analyses with specific markers of ATII (pro-SPC, Figure 7A) and ATI (AQP5, Figure 7B) cells in lung sections from WT and KO mice exposed or not to bleomycin. As shown in the representative images (top panel) and intensity quantifications (bottom panel), KO/PBS mice exhibit an increase in pro-SPC compared to WT/PBS mice. Bleomycin did not significantly affect the intensity of pro-SPC in either WT or KO mice, with the latter (KO/Bleo) remaining significantly higher than in the WT/Bleo (Figure 7A).

Similarly, significantly higher AQP5 levels were observed in KO than in WT mice, under physiological conditions (PBS, Figure 7B). In contrast to AT2 cell staining, which was unaffected after bleomycin-induced lung injury, a significant decrease in the intensity of the marker (AQP5) of the more fragile AT1 cells was observed in WT/Bleo and KO/Bleo after bleomycin challenge (23.3% and 23.7% decrease, respectively, compared to their respective controls, WT/PBS and KO/PBS). However, AQP5 remained significantly higher in KO/Bleo than in WT/Bleo mice.

### 3.7 Comparison of wound repair capacity of primary cultures of alveolar epithelial cell cultures from WT and KvLQT1-KO mice

**FIGURE 8:**
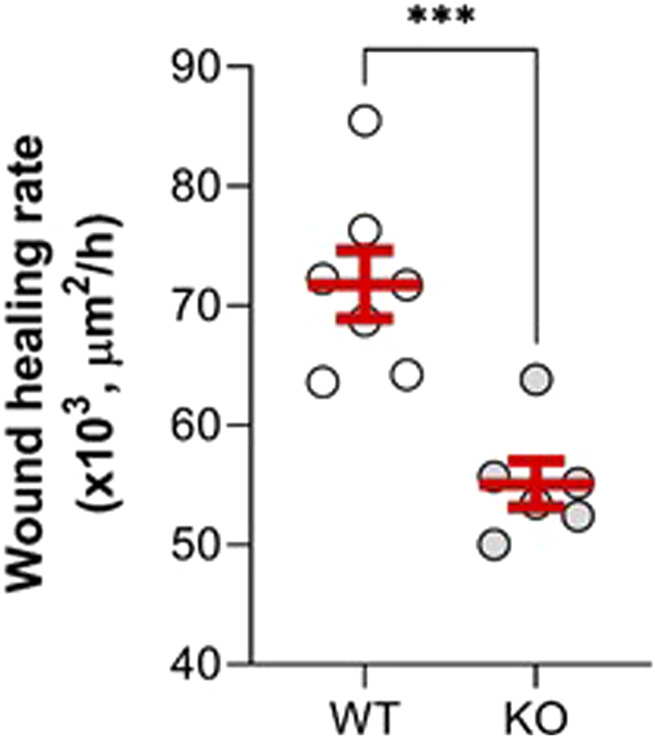
Repair rates of alveolar cell monolayers from WT and KvLQT1-KO mice. Wound-healing rates after mechanical injury of primary cultures (day 4) of alveolar cells isolated from the lungs of WT (n = 7) and KvLQT1-KO mice (n = 6) were monitored over a period of 6 h and plotted individually (each dot represents one alveolar cell culture). Images were taken at t = 0 and t = 6 h to evaluate the area of repair and then to calculate the wound-healing rates. Values are mean ± SEM. Unpaired *t*-test was performed. ***p < 0.001.

Our next goal was to evaluate the effect of KvLQT1 KO on the repair capacity of ATII cells, which are progenitor cells responsible for alveolar self-renewal and repair after injury. We therefore performed wound-healing assays to determine the specific contribution of the KvLQT1 channel, by comparing the repair rates of primary alveolar epithelial cell cultures, enriched for ATII cells obtained from the lungs of WT and KO mice. We found that alveolar cell cultures from KO mice had significantly lower wound healing rates after 6 h than those from WT mice (Figure 8), indicating that KvLQT1 function is essential for optimal early repair processes after injury.

## 4 Discussion

Our data first confirmed that the KvLQT1 potassium (K^+^) channel knockout had no major effect on parameters measured under physiological conditions (KO/PBS). As expected, bleomycin challenge in WT mice reproduced the key features of the acute exudative phase of ALI/ARDS (on day 7), with impaired respiratory function as well as an alteration of the alveolar-capillary barrier favoring infiltration of protein-rich fluid into the alveolar space, development of an inflammatory response, and histological evidence of heterogeneous damage including large areas of severe diffuse alveolar inflammatory damage. KvLQT1 deletion exacerbated the bleomycin-induced decline in lung function. Further analysis showed that the KCNQ1 KO did not alter the edema index, but modified the profile of infiltrating immune cells and reduced lung tissue inflammation, as observed in histological sections. Finally, a reduced repair rate of the KvLQT1-KO alveolar cells was observed. Collectively, these data suggest a complex role of KvLQT1 in lung homeostasis during the development and/or the resolution of acute phase parameters of ARDS in the bleomycin ALI model.

Molecular deletion of KvLQT1 in mice produces a Shaker-Waltzer phenotype, characterized by hyperactivity (head bobbing and intermittent bidirectional circling) and loss of balance (inner ear defects) (Casimiro et al., 2001). KvLQT1-KO mice are also known to exhibit macrocytic anemia, intestinal absorption defects and enhanced insulin sensitivity (Vallon et al., 2005; Boini et al., 2009). The effects of KCNQ1 deletion on cardiac function have been extensively studied by Dr. Pfeifer’s group, who described ECG abnormalities, particularly prolongation of the QTc and JT intervals and an increase in T- and P-wave area. These changes were associated with altered ventricular and atrial repolarization, but no differences in heart rate (RR interval). An increase in heart weight was also observed, but no changes in lung weight. There is no clear reason to suspect that the cardiac dysfunction could be responsible for the decline in lung compliance in KO/Bleo mice. Based on the similar water lung contents measured in WT/Bleo and KO/Bleo (Figure 4A), it is unlikely that a cardiogenic edema developed as a result of impaired heart function in KO mice, in addition to the alveolar lung edema secondary to bleomycin-induced ALI.

At baseline, our blood analysis results (Figure 1) showed no changes in electrolytes, consistent with Dr. Pfeifer’s observation (Vallon et al., 2005). No major differences in blood gases or metabolites were observed, except for significantly higher lactate levels in KO vs*.* WT mice under physiological conditions. It can be postulated that the observed hyperactivity of KO mice, due to a Shaker-Waltzer phenotype, is responsible for increased ATP demand and subsequent increased glycogenolysis/glycolysis by muscle cells, leading to this higher lactate production. The observed maintenance of glucose levels in KO/PBS mice is probably due to the homeostatic function of the liver with *de novo* glucogenesis from lactate.

Although the phenotype associated with KvLQT1-KO in mice has been described for various tissues (Casimiro et al., 2001; Vallon et al., 2005; Boini et al., 2009), the impact of KCNQ1 dysfunction in the lung was unknown until recently. In one of our previous studies (Aubin Vega et al., 2023), we confirmed effective *kcnq1* extinction in lung tissue, associated with a 50% decrease in total basolateral K^+^ current in respiratory epithelial cells from KO mice. In addition, no major impairment of lung function was observed at baseline, as confirmed in the present study (Figure 2; Figure 3). Furthermore, no impairment of the alveolar structure was observed in KCNQ1-KO mice under physiological conditions (Figure 6A). Healthy alveolar architecture has also been reported despite deletion of the regulatory (β) subunit of the KvLQT1 K^+^ channel, KCNE2 (Zhou et al., 2019) or KCNE3 (Preston et al., 2010).

Our search for early signs of respiratory and/or multi-organ failure, as often observed in patients with severe ARDS (Kallet et al., 2019), revealed some dysregulation of blood parameters in WT mice under pathological conditions. Bleomycin caused a simultaneous increase in pCO_2_ and HCO_3_^−^ in both WT/Bleo and KO/Bleo mice, although no change in pH was observed. The rise in pCO_2,_ which is relative to ineffective gas exchange, is consistent with other studies using this experimental ALI model (Shi et al., 2019; Miyamoto et al., 2022). However, our results for pH level do not replicate the changes observed in other studies using bleomycin (Shi et al., 2019; Miyamoto et al., 2022). These divergent data may be explained by the different compartments from which the blood was collected (arterial in the literature vs. venous in our study) (Shi et al., 2019; Miyamoto et al., 2022). Regarding metabolites, the marked decrease in blood glucose in WT/Bleo mice compared to their control littermates (WT/PBS) is likely related to reduced food intake as a result of bleomycin-induced debilitation (Cowley et al., 2019). In addition, a loss of (fat and muscle) mass with reduced physical activity may be related to the observed decrease in lactate levels, in both WT/Bleo and KO/Bleo mice. We also showed that the lactate levels remained higher in KO/Bleo than in WT/Bleo, thus allowing glucose levels in KO/Bleo mice to be maintained by hepatic gluconeogenesis. Our measurements also showed that the KvLQT1 deletion did not exacerbate the systemic changes induced by bleomycin. Actually, no warning signs of multiorgan failure were observed in bleomycin-treated animals (WT/Bleo and KO/Bleo), possibly because of the experimental conditions, in particular the mode of bleomycin administration (intranasal instillation, which minimizes extrapulmonary damage, compared to systemic injection), the chosen protective dose of bleomycin (which caused sufficient lung damage without mortality), and the duration of the experiment (which focused on the acute phase of ALI, i.e., day 7).

Assessment of respiratory function in both WT and KO mice (Figure 2; Figure 3) revealed that bleomycin caused an alteration in three important lung mechanical properties, namely, resistance, elastance and compliance. The findings of the present study are consistent with the literature, more specifically with those of two articles that reported a decrease in compliance in bleomycin-exposed animals (Shi et al., 2019; Miyamoto et al., 2022). We found a pronounced shift in the pressure-volume curve of KO/Bleo mice, and a concomitant decrease in compliance compared with WT/Bleo mice, as well as a significant deterioration in their respiratory system resistance and elastance. This is the first time that a worsening of these parameters, caused by KvLQT1 depletion has been demonstrated, although impairment of lung function after other types of K^+^ depletion has already been reported. Indeed, Schwingshackl *et al.* showed that genetically engineered mice deficient in the K2P K^+^ channels TREK-1 (Schwingshackl et al., 2014) or TREK-1/TREK-2/TRAAK (Schwingshackl et al., 2017) exhibited altered lung compliance compared to the WT mice after a short period of hypoxia followed by mechanical ventilation (double insult model).

Evaluation of the alveolar-capillary barrier function (Figure 4) showed lower endothelial permeability in KO/Bleo than in WT/Bleo mice. The mRNA expression of KCNQ1 to KCNQ5 subunits has been reported in coronary vascular tissue, but it has not been clearly defined whether KvLQT1 were expressed in smooth muscle and/or other cell types such as the endothelium (Goodwill et al., 2016). There is evidence for Kv7 expression in airway smooth cells (Brueggemann et al., 2012); however, to our knowledge, the presence of KCNQ1/Kv7.1 channels in alveolar capillary endothelial cells has not been clearly demonstrated. Although it would have been interesting to study the role of KvLQT1 channels in the endothelial compartment, we focused our study mainly on the alveolar epithelium because of its critical role in the resolution of ALI, especially in the bleomycin model, which induces more alveolar than interstitial damage. Despite reduced endothelial permeability in KO/Bleo than in WT\Bleo mice, both WT and KO mice developed comparable protein-rich pulmonary edema (with similar levels of water lung and BALF protein contents) after exposure to bleomycin. A compensatory role of other types of K^+^ channels expressed in respiratory epithelial cells (Bardou et al., 2009), which could participate to the control of ion and fluid absorption, can be postulated.

In bleomycin-exposed WT mice, we observed an increase in total immune cell numbers, mainly due to massive neutrophil infiltration, and elevated levels of pro-inflammatory cytokines/chemokines (Figure 5). Massive neutrophilic infiltration is a known phenomenon in patients with ARDS that is well reproduced in this experimental model (Matthay et al., 2019). While KO/Bleo mice had the same total number of infiltrating immune cells in BAL as WT/Bleo mice, we observed an alteration of the immune cell profile in KO/Bleo, with a lower neutrophil/macrophage ratio, while the lymphocyte levels were similar in WT/Bleo and KO/Bleo mice. Histological sections showed less congestion, while qPCR measurements indicated lower cytokine/chemokine expression levels in the total lung tissue compared to WT/Bleo mice. According to the literature, whether under physiological or pathological conditions, the effect of K^+^ channel deletion on the pulmonary inflammatory response depends on the types of K^+^ channels or the experimental conditions (i.e., as a function of the animal model or the time point chosen) (Schwingshackl et al., 2014; Zhou et al., 2019; Vega et al., 2020). In contrast to the present data, KCNE2-deficient mice showed elevated levels of BAL TNF-α and IL-6 compared to WT mice, under basal conditions. In TREK-1 shRNA-deficient alveolar cells exposed to hyperoxia (Schwingshackl et al., 2012), a rapid (48 h) increase in MCP-1 levels was demonstrated, whereas no changes in IL-6 and KC were observed, similar to the results of this study.

The mechanism by which KvLQT1 silencing leads to an altered immune cell profile in the presence of lung damage remains to be defined, but several hypotheses could be proposed. First, the lower endothelial permeability in KO/Bleo mice than in WT/Bleo mice, as indicated by the Evans Blue extravasation assay, could physically impede neutrophil migration. Lower levels of cytokines/chemokines acting as neutrophil chemoattractants combined with a better preserved endothelial barrier is another possible explanation for the reduced neutrophil/macrophage ratio. This hypothesis is supported by the findings of Meduri *et al.* who described in a clinical study that the reduction of BAL inflammatory cytokines was associated with an improvement in endothelial permeability indices (Meduri et al., 1995b). Furthermore, Immler *et al.* demonstrated that neutrophils lacking another type of voltage-dependant K^+^ channel, Kv1.3, lose their ability to adhere and transmigrate to the alveolar lumen (Immler et al., 2022). Finally, pharmacological modulation of large conductance K^+^ (BK) channels alters cytokine secretion by human pulmonary endothelial cells (Zyrianova et al., 2023). These potential mechanisms of action may partially explain our findings, but the impact of the KvLQT1 modulation on the inflammatory response requires further investigation.

We confirmed the presence of severe epithelial inflammatory damage in the lungs of WT mice during the acute phase after bleomycin challenge (Figure 6). We observed heterogeneous inflammatory lesions and different degrees of severity depending on the lung area (with zones of healthy structure and/or inflammation). The main features observed in histological sections were cellular infiltration, congestion and thickening of the septa, culminating in deformation of the alveolar structure. KO/Bleo mice appeared to develop significantly less inflammatory damage, as indicated by the ratio of injured/inflamed parenchymal area, which remained as low as the control group (KO/PBS), or the lung injury score, which was significantly lower compared to WT/Bleo mice. Nevertheless, we noticed a significant 23% decrease in AQP5 intensity (Figure 7) in both WT/Bleo and KO/Bleo groups, suggesting that epithelial ATI cells, which are known to be fragile, were injured in the two experimental groups. In contrast, the intensity of the ATII marker pro-SPC was not altered after bleomycin exposure.

Based on our analyses of the alveolar-capillary barrier, inflammatory response and histological observation, it is questionable why the impairment of lung function after bleomycin-induced ALI is worse in KO than in WT mice. Several hypotheses could have been proposed to explain the observed aggravation of the decline in lung compliance and the concomitant increase in resistance. An exacerbation of pulmonary edema in KO/Bleo mice could have been responsible for the loss of lung function. However, the similar water lung content measured in WT/Bleo and KO/Bleo mice does not support this hypothesis. A deficiency in surfactant production could also be accountable for the altered compliance. Although the observed high levels of pro-SPC in KO mice seem to contradict this hypothesis, it cannot be completely ruled out as the actual levels of surfactant released are not known. Finally, the degree of the lung function impairment may be related to lung damage severity. However, our histological analyses showed no worsening of the injury scores in KO/bleo compared to WT/Bleo mice. A lack of correlation between lung injury scores and function decline has been reported in other studies. In a bleomycin model, Manali et al. (Manali et al., 2011) observed that lung elastance (H) and lung PV loop curve area did not correlate strongly with histological scores. Furthermore, Toumpanakis et al. (Toumpanakis et al., 2023) showed that a severe lung injury induced by direct LPS inhalation was not associated with deterioration in lung compliance and resistance.

Finally, we observed a slowdown of the repair rates after injury of KvLQT1-KO alveolar cells in the wound-healing assays (Figure 8). In a previous study from our laboratory (Trinh et al., 2007), we investigated the functional relationship between K^+^ channels, specifically KvLQT1, and growth factor signaling during alveolar epithelial migration, proliferation, and repair. There is also evidence for a possible interaction between other types of K^+^ channels and integrins or focal adhesion kinase, both of which are key players in the epithelial cell migration process (Wei et al., 2009; Girault et al., 2015; Schwingshackl et al., 2015). However, the precise mechanisms by which KvLQT1 is involved in the control of alveolar repair processes would require further investigation.

In conclusion, we used a representative *in vivo* model of ALI that mimics specific features of ARDS, such as severe lung damage, a neutrophilic inflammatory response, protein-rich fluid infiltration, and deterioration of lung compliance, to study the role of the K^+^ channel KvLQT1, in the development and resolution of the acute phase of ARDS. Our results suggest the involvement of KvLQT1 K^+^ channels in the modulation of biophysical properties of lung function, the response of immune cells in an inflammatory context, and the regulation of the epithelial repair process. These findings are of interest for a deeper understanding of the role of K^+^ channels in the pathophysiology and resolution of ARDS as well as for the development of new therapeutic strategies.

## Data Availability

The raw data supporting the conclusion of this article will be made available by the authors, without undue reservation.
