# Supplementary material for: Function of KvLQT1 potassium channels in a mouse model of bleomycin-induced acute lung injury
Source: Front Physiol. 2024 Feb 20;15:1345488. doi: 10.3389/fphys.2024.1345488 (PMC10912346; doi:10.3389/fphys.2024.1345488)
Supplement: Supplementary file 1 [file Presentation1.pdf]

Supplementary Material - Supplementary Figures

Supplementary Figure 1

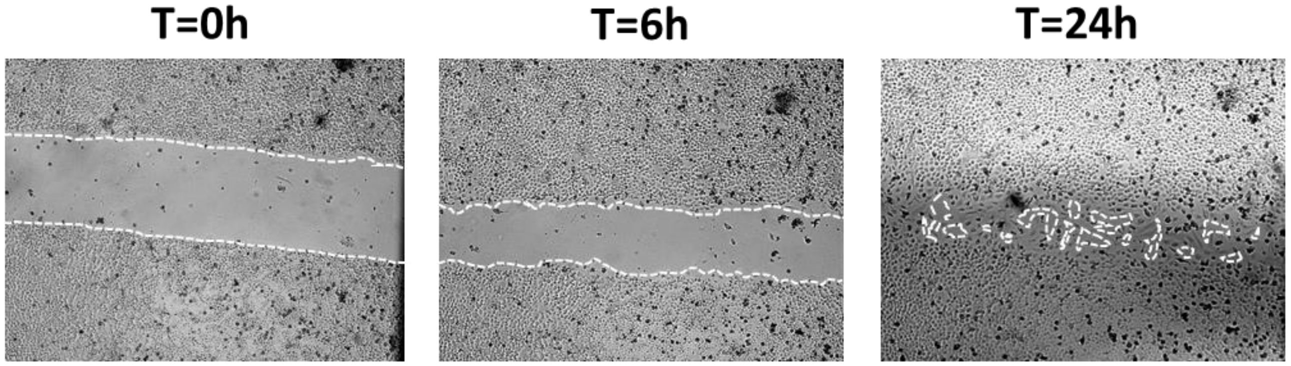

<u>Supplementary Figure 1:</u> Wound-healing assay. Representative images of primary cultures (day 4) from mouse alveolar cells after mechanical injury (time 0) and after 6h and 24 h of repair. Dotted lines have been added to the images along the wound edges to illustrate how the wound areas are defined at T0 and T6 of repair.
